# Supplementary material for: Formulation, Preparation, Characterization, and Evaluation of Dicarboxylic Ionic Liquid Donepezil Transdermal Patches
Source: Pharmaceutics. 2022 Jan 16;14(1):205. doi: 10.3390/pharmaceutics14010205 (PMC8812279; doi:10.3390/pharmaceutics14010205)
Supplement: Supplementary file 1 [file pharmaceutics-14-00205-s001.zip › pharmaceutics-1525849-supp-for confirmation.pdf]

Supplementary Materials: Formulation, Preparation, Characterization, and Evaluation of Dicarboxylic Ionic Liquid Donepezil Transdermal Patches

Linh Dinh, Soohun Lee, Sharif Md Abuzar, Heejun Park and Sung-Joo Hwang

Standard 1H NMR Chloroform-D (D, 99.8%) + 0.05 % TMS + silver foil (Cambridge Isotope Laboratories, Andover, MA, USA) solvent

1H NMR spectra of DPZ ILs

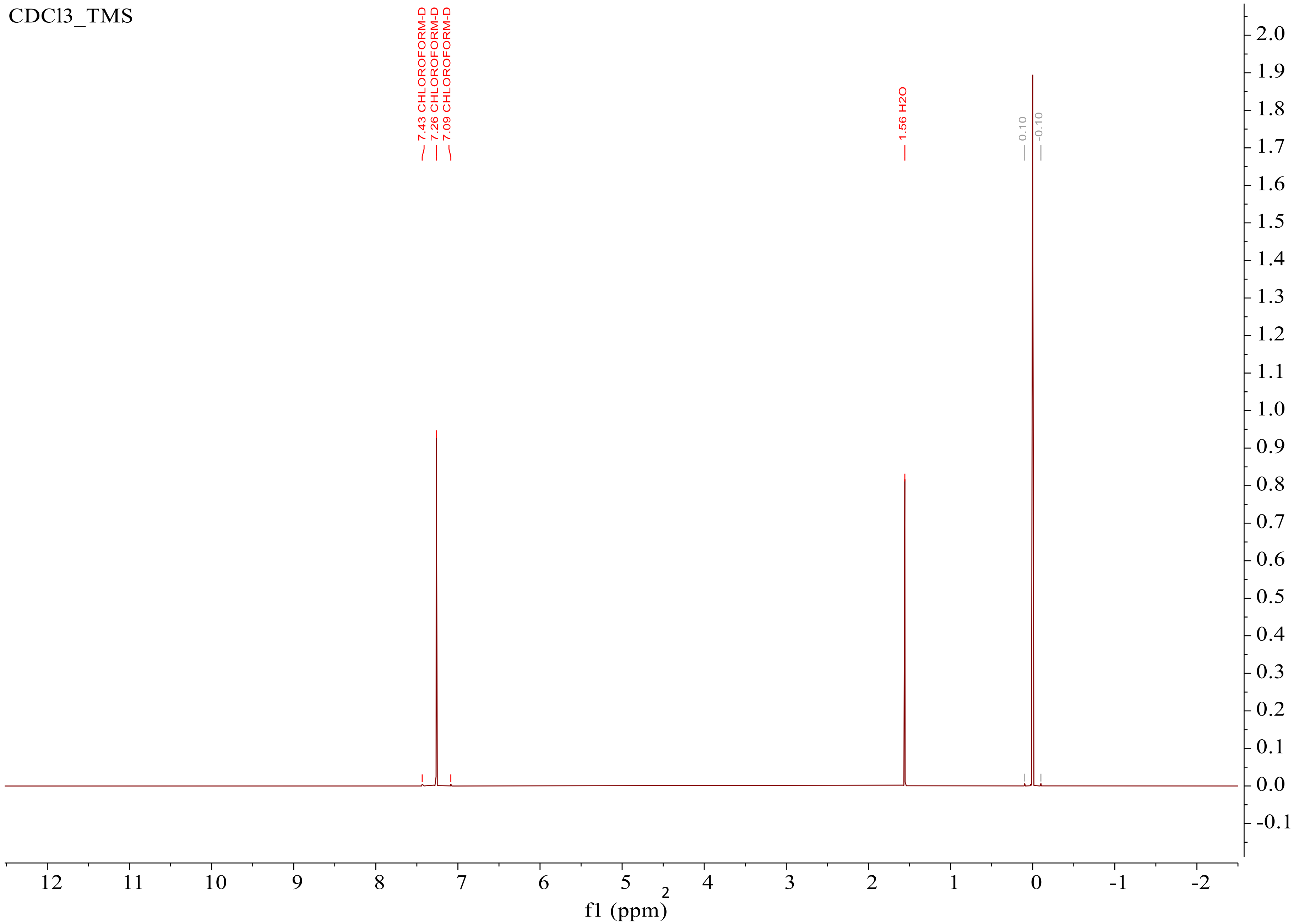

CDCl3_TMS
7.43 CHLOROFORM-D
7.26 CHLOROFORM-D
7.09 CHLOROFORM-D
1.56 H2O
0.10
-0.10
f1 (ppm)

Figure S1. 1H NMR spectrum of DPZ-adipic acid IL. 1H NMR (600 MHz, CHLOROFORM-D) δ 7.31 – 7.20 (m, 5H), 7.19 (s, 2H), 7.09 (s, 1H), 6.78 (s, 1H), 3.89 (s, 3H), 3.83 (s, 3H), 3.65 (q, J = 7.1 Hz, 4H), 3.20 – 3.13 (m, 1H), 3.06 – 2.98 (m, 2H), 2.66 – 2.58 (m, 2H), 2.35 – 2.24 (m, 2H), 2.12 (d, J = 10.6 Hz, 1H), 2.08 (d, J = 10.8 Hz, 1H), 1.87 – 1.77 (m, 1H), 1.72 – 1.65 (m, 4H), 1.46 – 1.33 (m, 3H), 1.33 – 1.22 (m, 2H), 1.18 (t, J = 7.0 Hz, 6H), 0.84 – 0.74 (m, 2H).

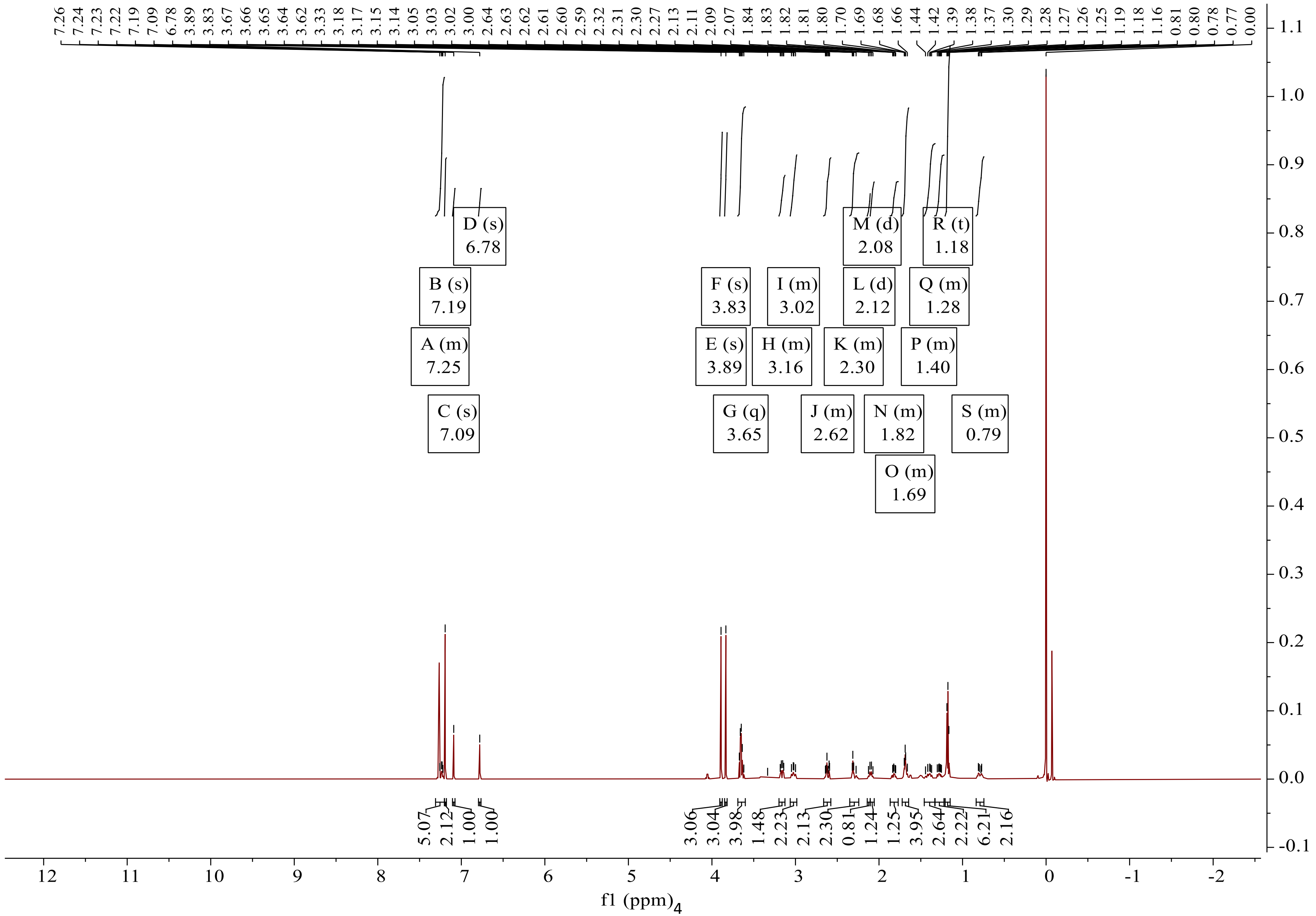

DPZ-adipic acid IL

Figure S2. 1H NMR spectrum of DPZ-azelaic acid IL.

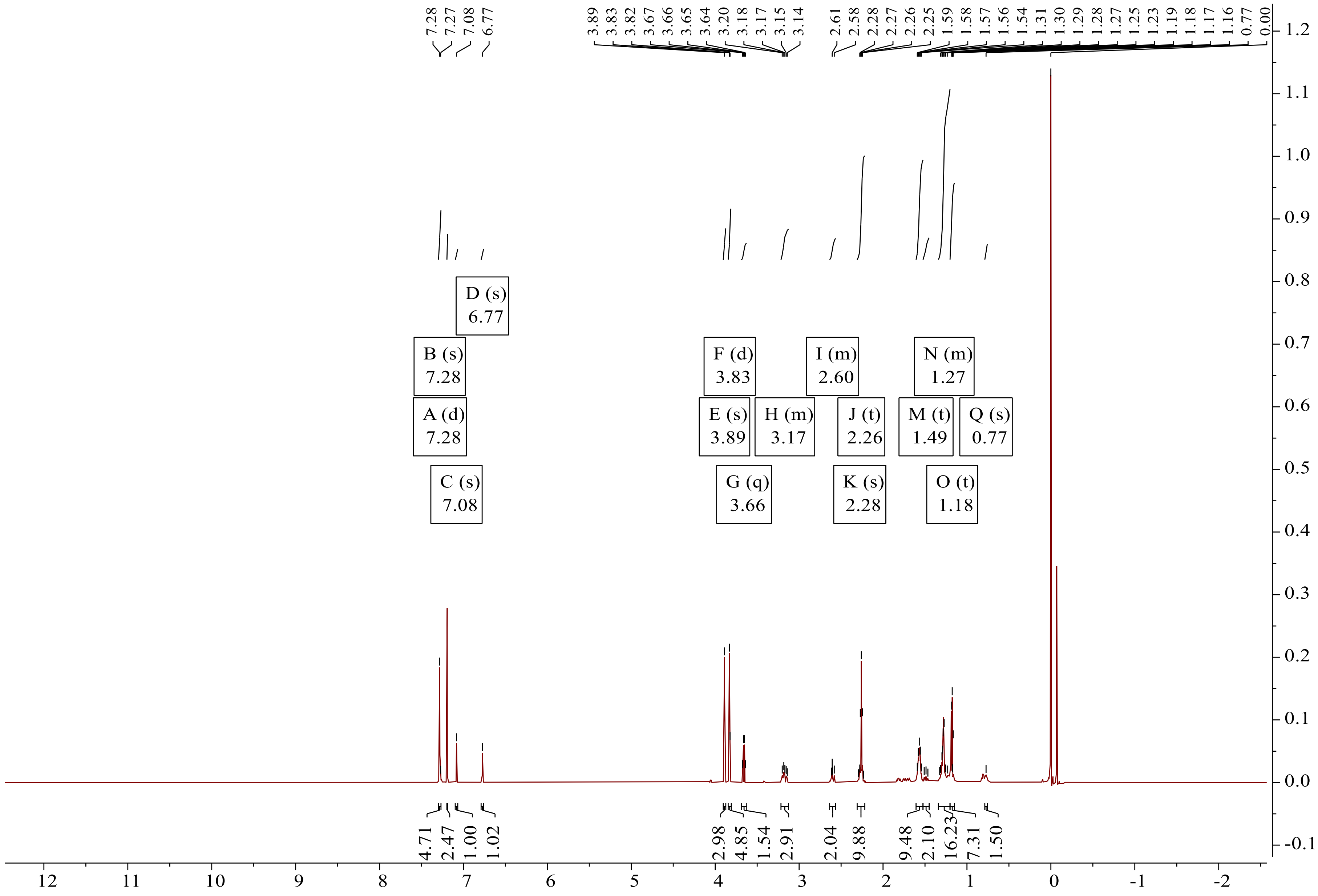

DPZ-azelaic acid IL

Figure S3. 1H NMR spectrum of DPZ-glutaric acid IL.

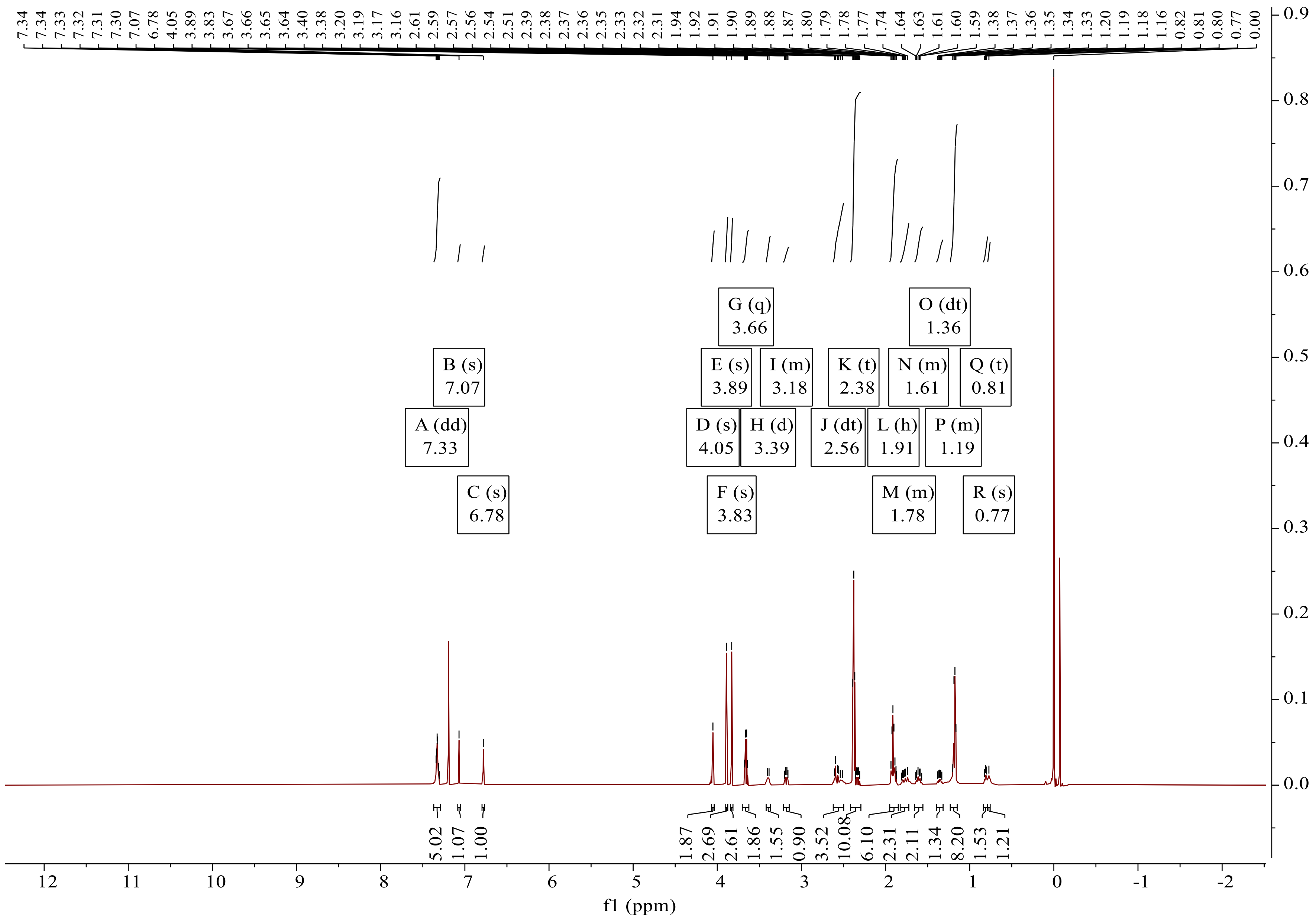

DPZ-glutaric acid IL

Figure S4. 1H NMR spectrum of DPZ-itaconic acid IL.

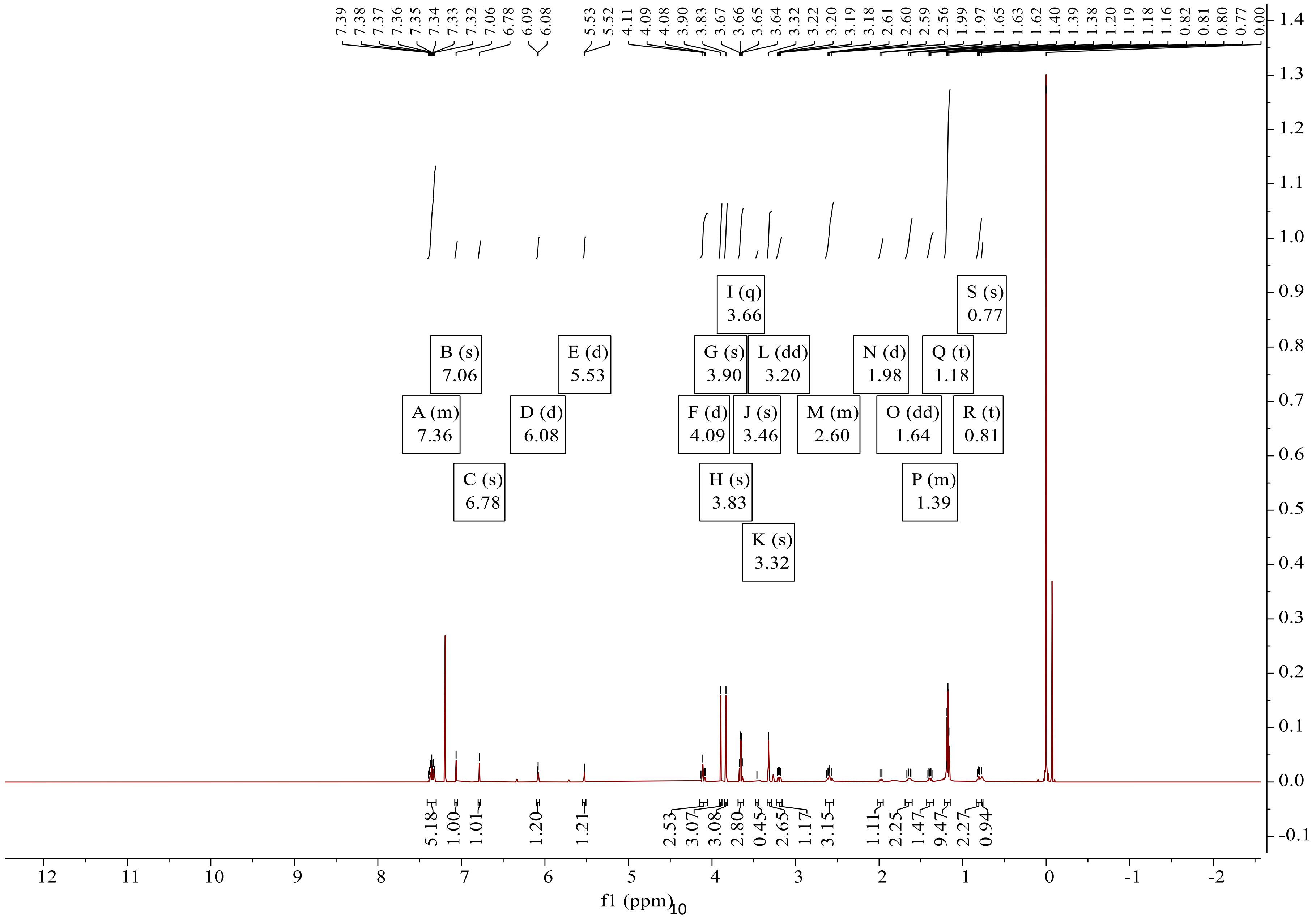

DPZ-itaconic acid IL

Figure S5. 1H NMR spectrum of DPZ-maleic acid IL.

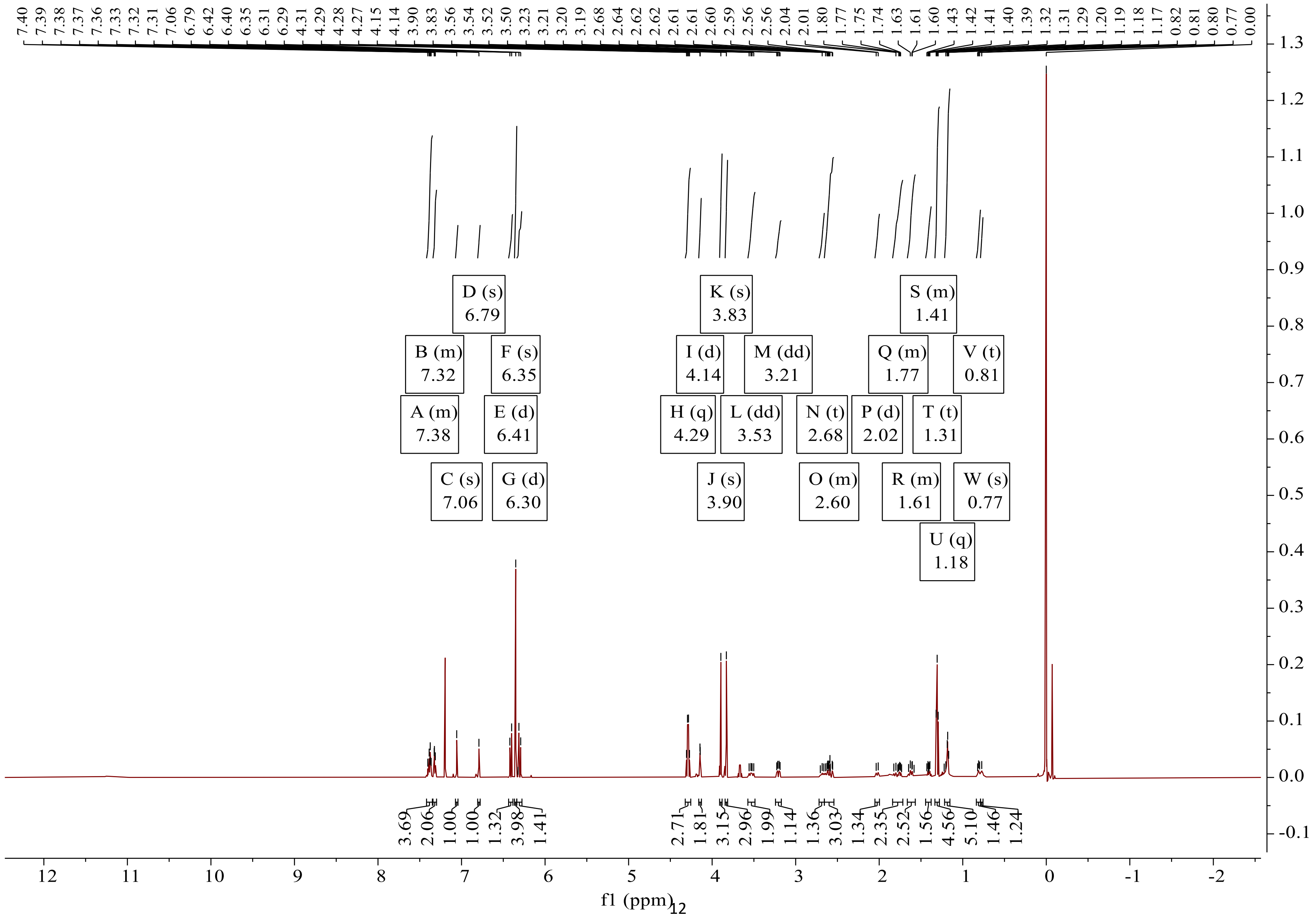

DPZ-maleic acid IL

Figure S6. 1H NMR spectrum of DPZ-malic acid IL.

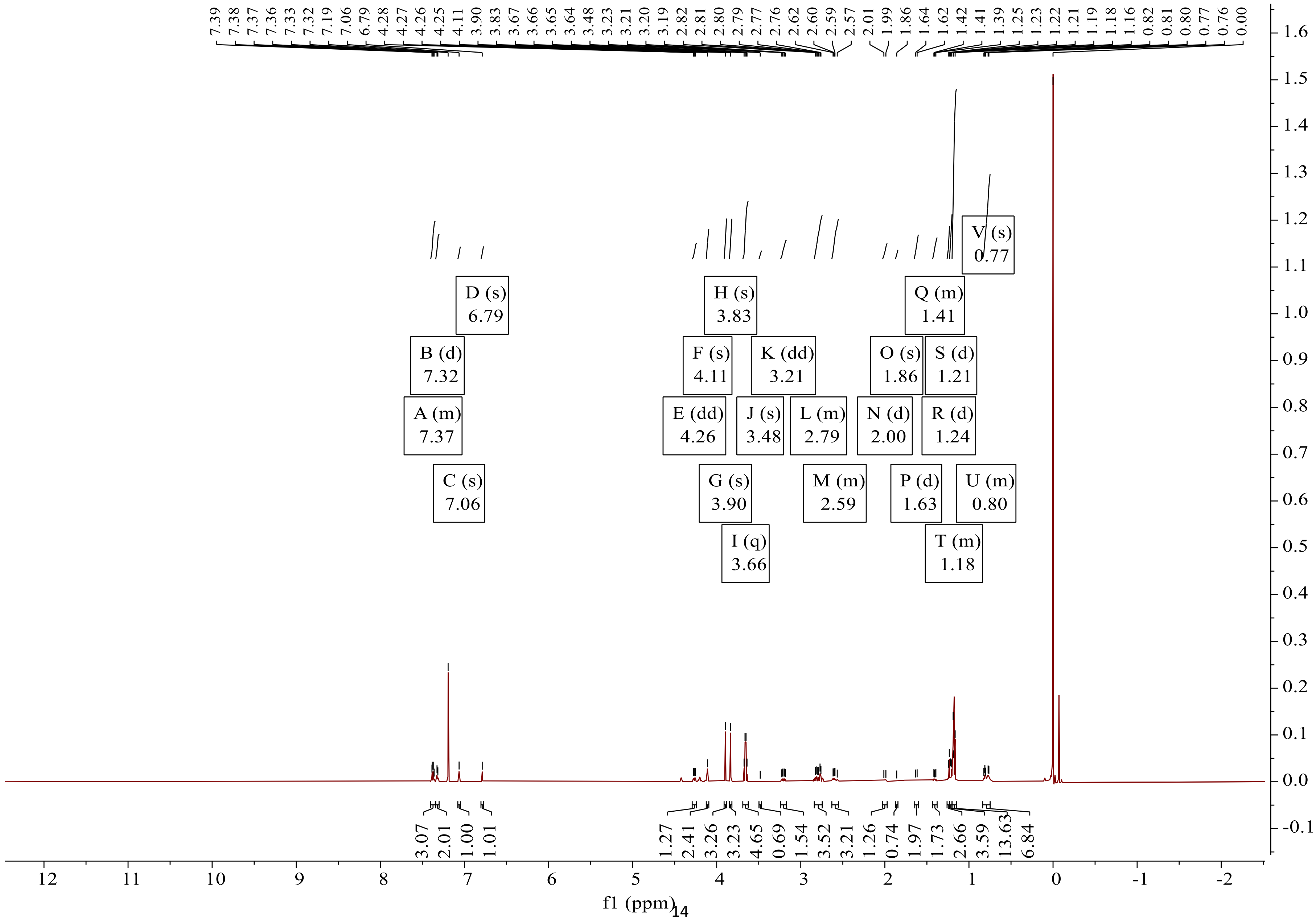

DPZ-malic acid IL

Figure S7. 1H NMR spectrum of DPZ-phthalic acid IL.

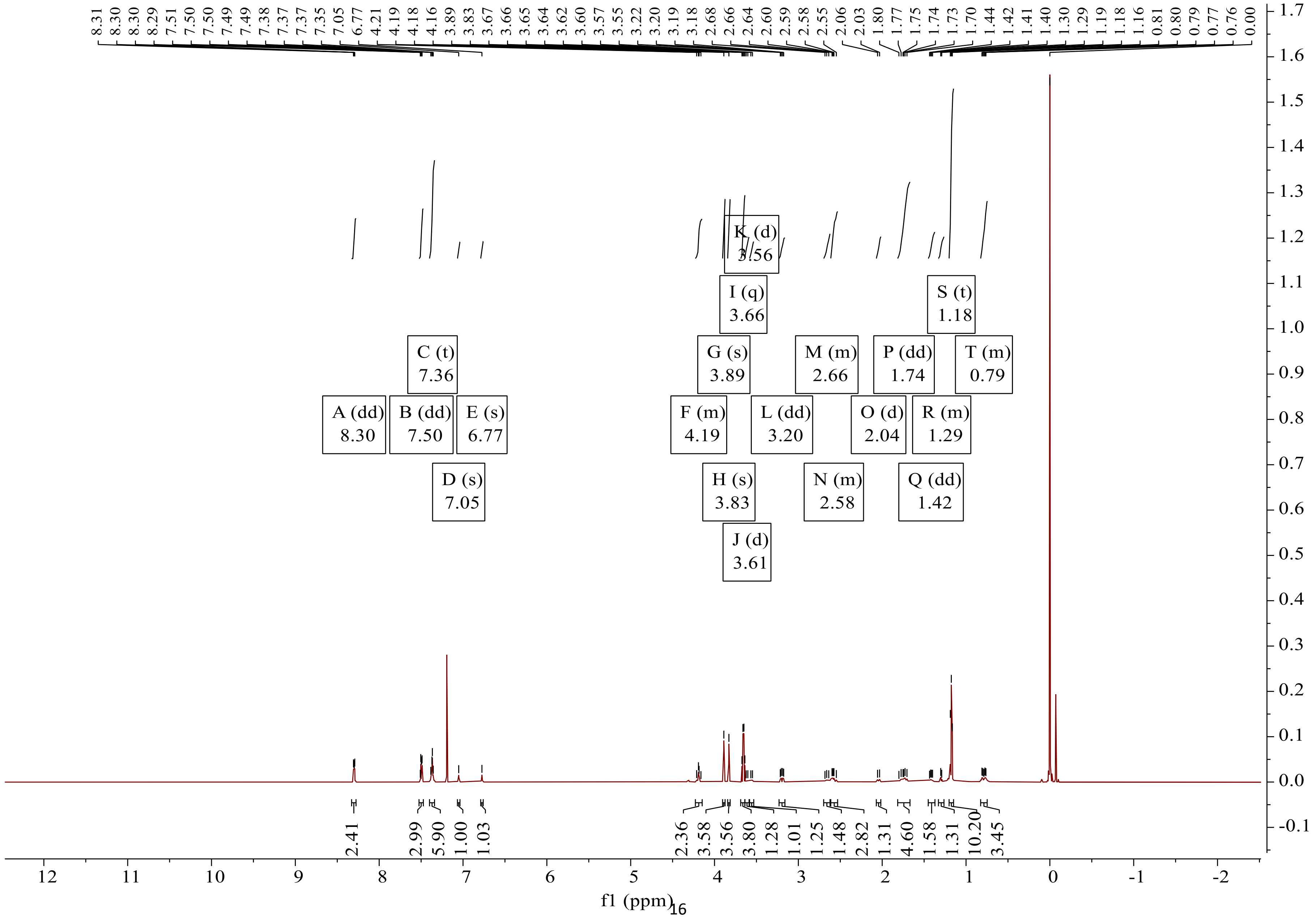

DPZ-phthalic acid IL

Figure S8. 1H NMR spectrum of DPZ-pimelic acid IL.

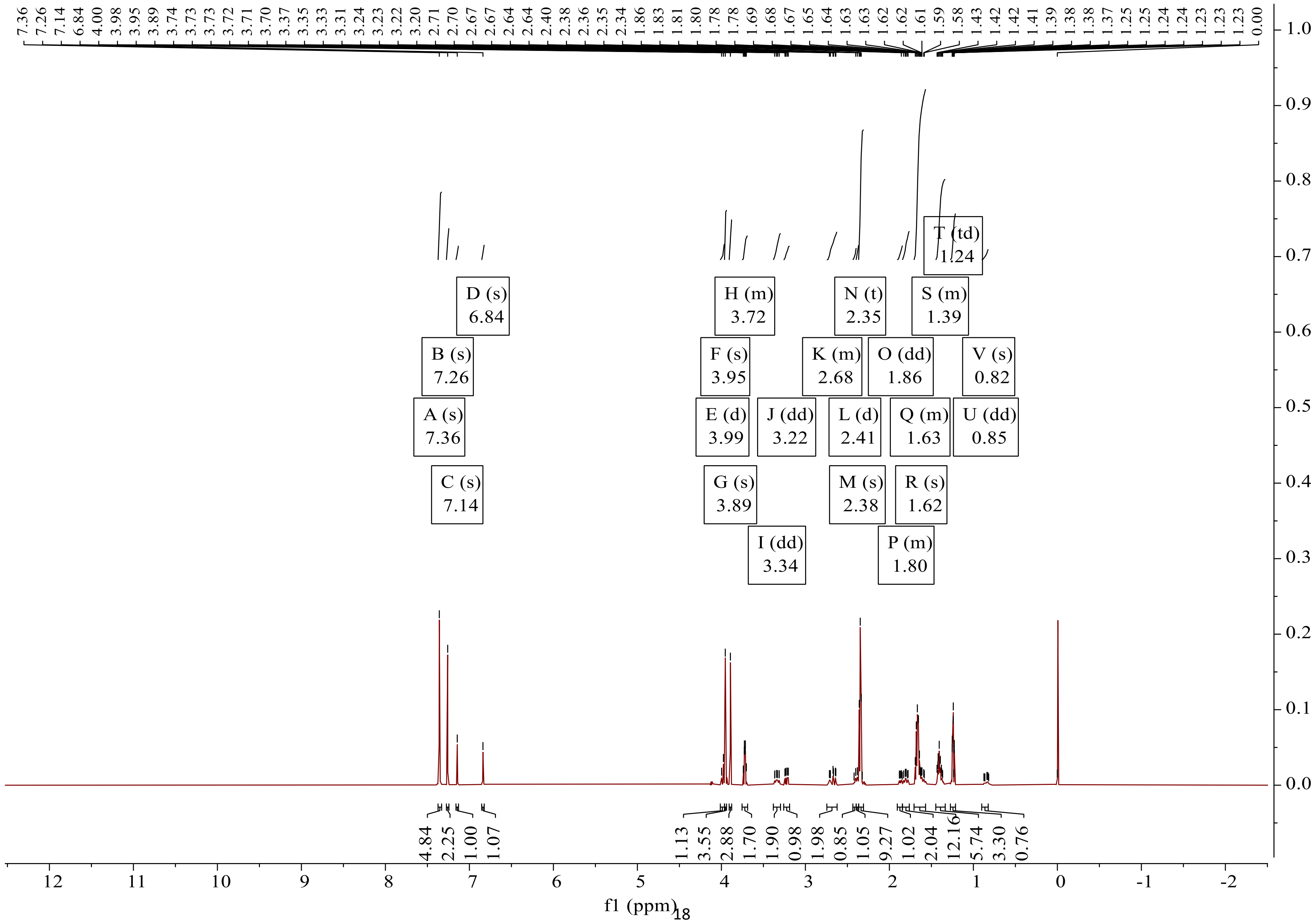

DPZ-pimelic acid IL

Figure S9. 1H NMR spectrum of DPZ-sebacic acid IL.

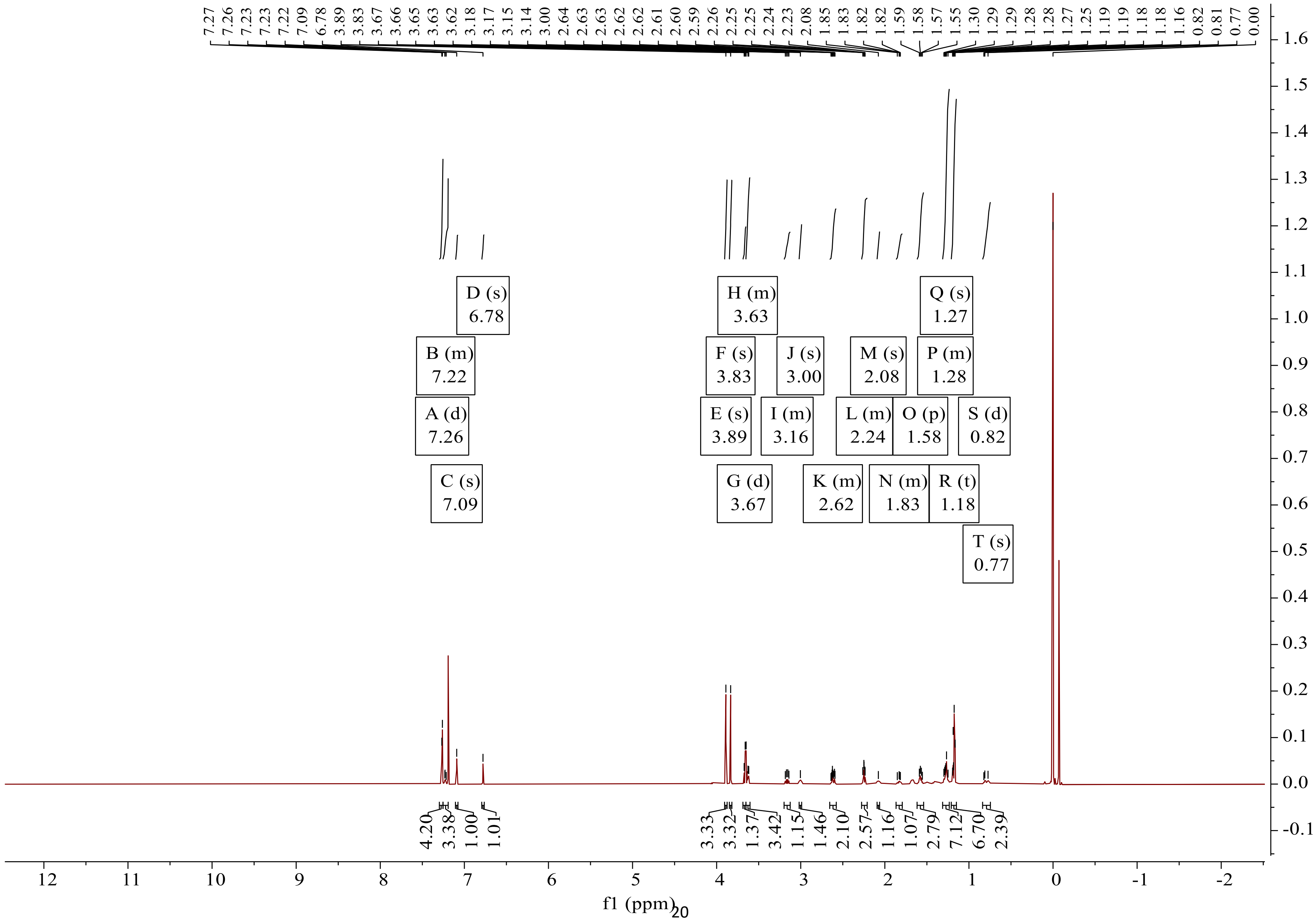

DPZ-sebacic acid IL

Figure S10. 1H NMR spectrum of DPZ-suberic acid IL.

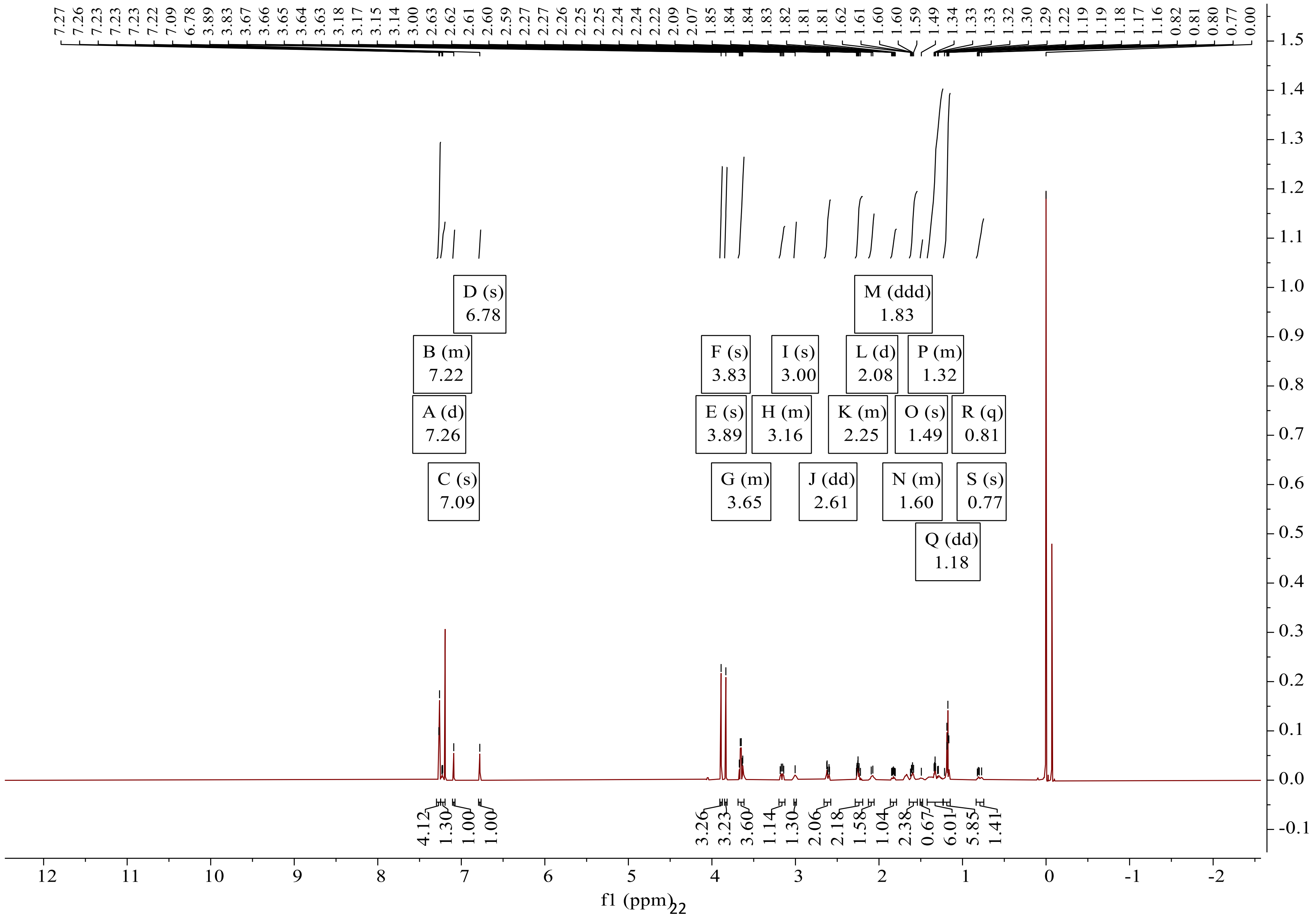

DPZ-suberic acid IL

Figure S11. 1H NMR spectrum of DPZ-succinic acid IL.

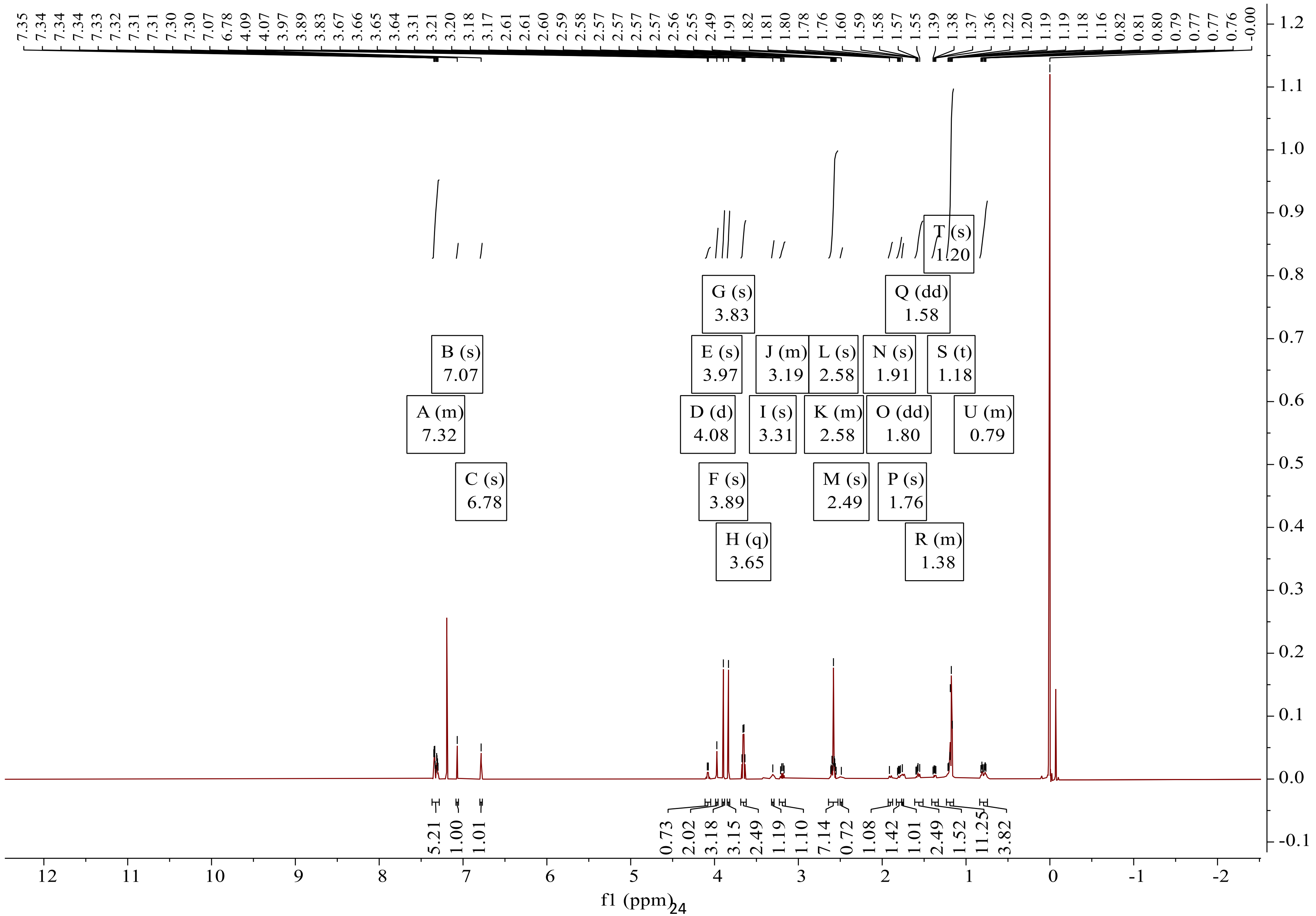

DPZ-succinic acid IL

Figure S12. 1H NMR spectrum of DPZ-tartaric acid IL.

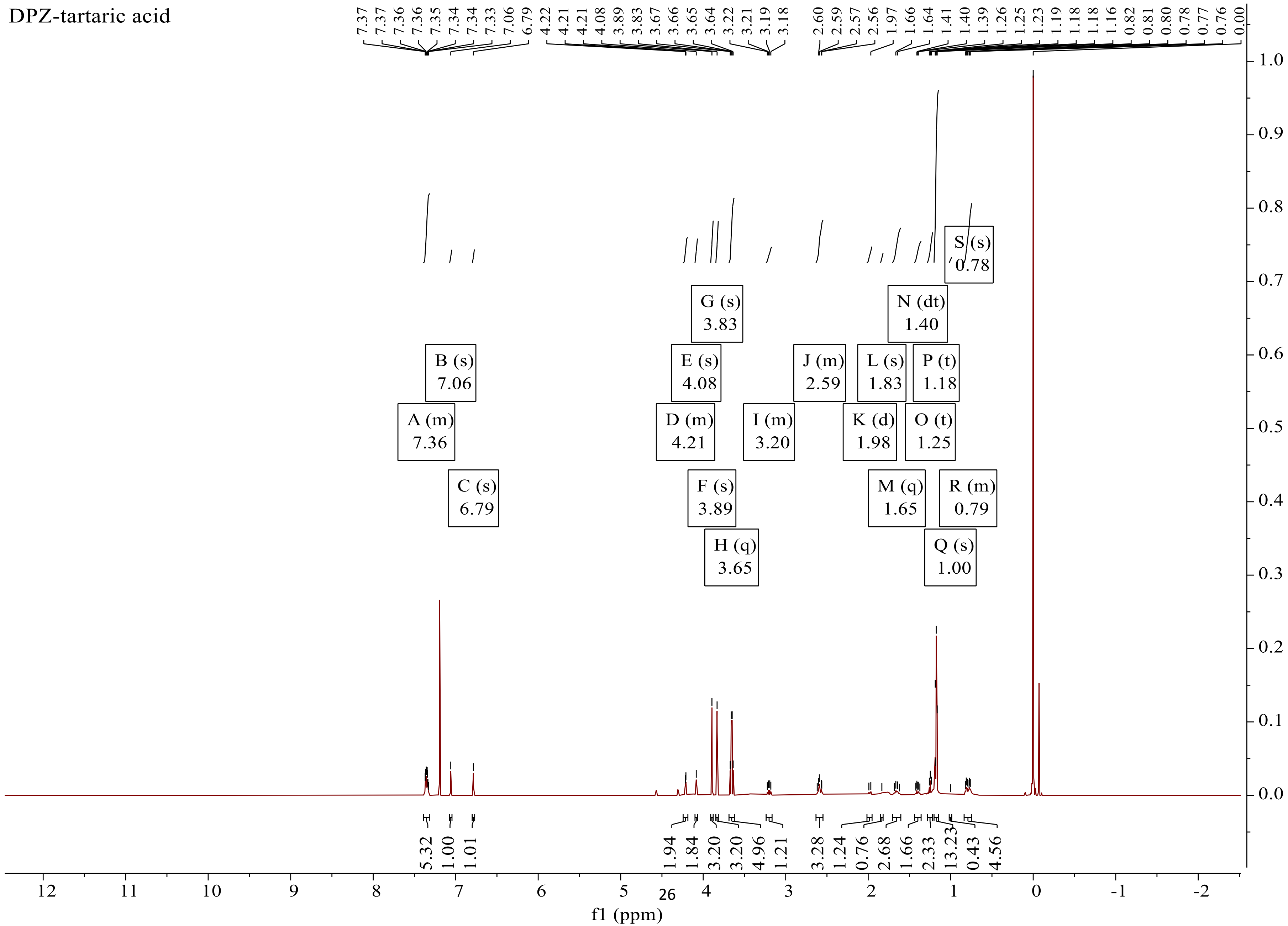
DPZ-tartaric acid
7.37
7.37
7.36
7.36
7.35
7.34
7.34
7.33
7.06
6.79
4.22
4.21
4.21
4.08
3.89
3.83
3.67
3.66
3.65
3.64
3.22
3.21
3.19
3.18
2.60
2.59
2.57
2.56
1.97
1.66
1.64
1.41
1.40
1.39
1.26
1.25
1.23
1.19
1.18
1.18
1.16
0.82
0.81
0.80
0.78
0.77
0.76
0.00
A (m) 7.36
B (s) 7.06
C (s) 6.79
D (m) 4.21
E (s) 4.08
F (s) 3.89
G (s) 3.83
H (q) 3.65
I (m) 3.20
J (m) 2.59
K (d) 1.98
L (s) 1.83
M (q) 1.65
N (dt) 1.40
O (t) 1.25
P (t) 1.18
Q (s) 1.00
R (m) 0.79
S (s) 0.78
5.32
1.00
1.01
1.94
1.84
3.20
3.20
4.96
1.21
3.28
1.24
0.76
2.68
1.66
2.33
13.23
0.43
4.56
f1 (ppm)

Figure S13. 1H NMR spectrum of DPZ-alpha keto glutaric acid IL.

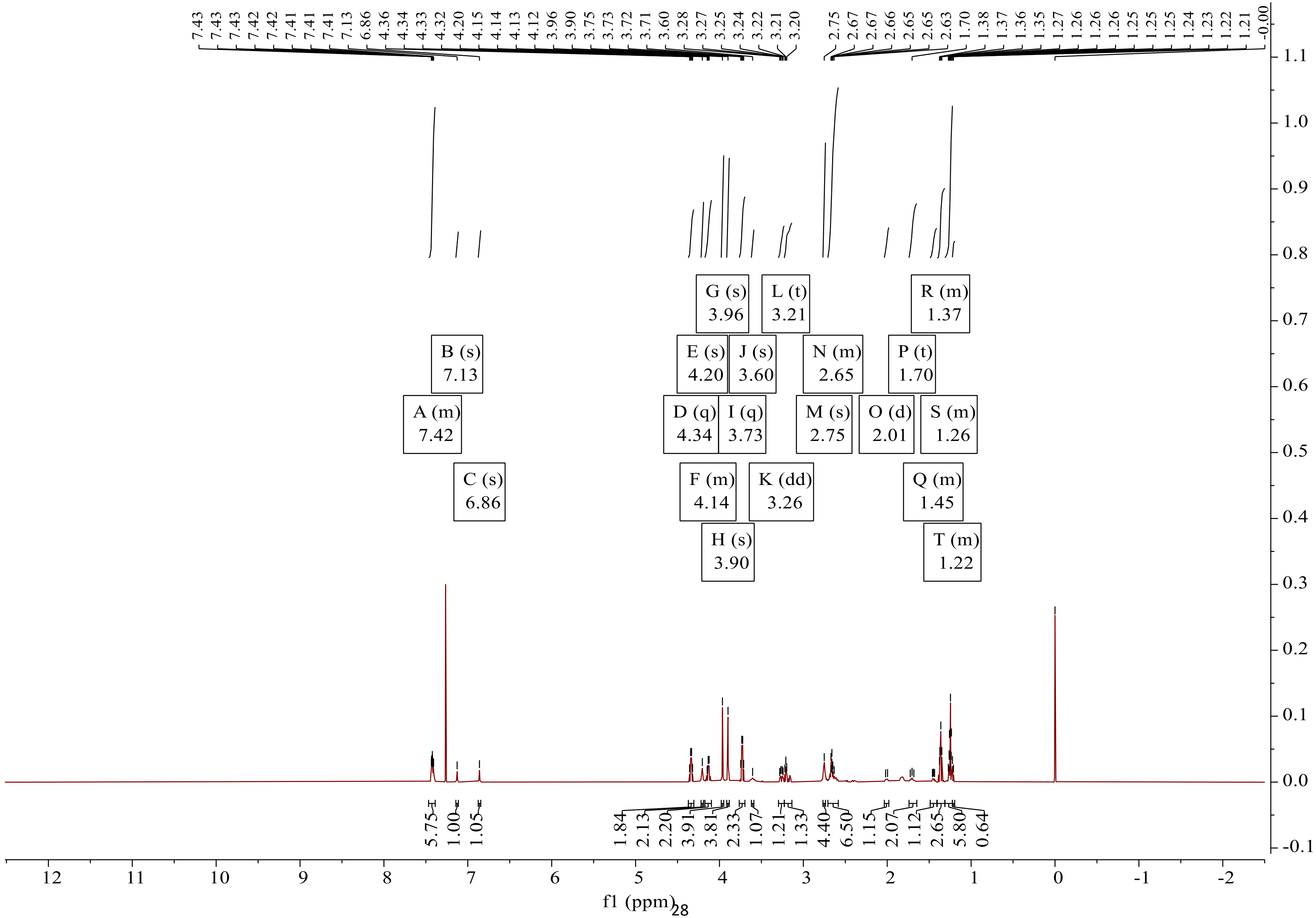

DPZ-alpha-keto glutaric
